# Patients’ experience of integrated nursing care in general practices in Switzerland: a mixed-methods study

**DOI:** 10.1093/fampra/cmaf011

**Published:** 2025-03-19

**Authors:** Muriel Schütz Leuthold, Fatima El Hakmaoui, Renzo Scuderi, Nicolas Senn, Christine Cohidon

**Affiliations:** Department of Family Medicine, Center for Primary Care and Public Health, University of Lausanne, Switzerland; Department of Family Medicine, Center for Primary Care and Public Health, University of Lausanne, Switzerland; Department of Family Medicine, Center for Primary Care and Public Health, University of Lausanne, Switzerland; Department of Family Medicine, Center for Primary Care and Public Health, University of Lausanne, Switzerland; Department of Family Medicine, Center for Primary Care and Public Health, University of Lausanne, Switzerland

**Keywords:** continuity of care, nursing, primary care, multidisciplinary care, case management

## Abstract

**Background:**

In many high-income countries, nurses, including registered nurses (RNs), play a key role in primary care (PC), particularly in general practice. Their involvement enhances patients’ experiences, especially in terms of accessibility and comprehensiveness of care provided. To reinforce the provision of care and enhance patients’ experience in family medicine, RNs were integrated into eight private general practices in the canton of Vaud, Switzerland, creating interprofessional teams. This study assessed patients’ experiences with new nursing activities in general practices.

**Methods:**

A mixed-methods approach was used to assess patients’ experiences. Quantitative data were collected through a patient experience survey conducted before and after nursing follow-up, with descriptive and bivariate analyses performed. Qualitative data were obtained from interviews with ten patients.

**Results:**

A total of 109 patients completed the questionnaire before and after nursing follow-up. Descriptive analyses showed that several dimensions of patients’ experience improved with new nursing follow-up. Bivariate analyses revealed significant improvements in several areas, including unmet healthcare needs, accessibility to nursing care, preventive care, and information provided. Furthermore, patients reported positive changes in their health and lifestyle due to preventive care. Qualitative data supported these results, highlighting the importance of nurses’ accessibility and availability and the holistic nursing care provided.

**Conclusion:**

These findings highlight the potential of nurse-led case management to address gaps in PC delivery, particularly in managing chronic diseases. The integration of nurses into general practice settings improved the provision of preventive care, enhanced patient education, and increased accessibility to care.

**Key messages**

- Nurses have significantly enhanced the provision of preventive care in general practices, facilitating improved prevention of health issues, and supporting the adoption of healthier lifestyle habits.
- Patients perceived the information provided by nurses as more comprehensive and exhaustive.
- Patients responded positively to the substitution of the general practitioner by the nurse.

## Introduction

Certain high-income countries have been involving nurses in primary care (PC) for many years, particularly in general practices. Thanks to the wide range of activities they can perform, nurses can significantly reinforce PC and enhance the overall efficiency of healthcare delivery [1–4], which is in ever greater demand due to ageing populations and the increasing prevalence of chronic illnesses. Nurses are the most represented profession in PC structures, highlighting their crucial role in ensuring comprehensive and accessible healthcare [5]. Depending on their level of training, nurses can play different roles and carry out different types of activities [1, 2]. In general practices, advanced practice nurses (APNs) generally play an autonomous role in medical screening, diagnosis, and prescribing medication [2, 6], whereas registered nurses (RNs) are generally more involved in traditional nursing activities and the management and follow-up of chronic patients [7–9].

In Switzerland, stronger interprofessional (IP) PC teams, including nurses, have recently emerged to address the anticipated shortage of general practitioners (GPs) in some regions [10]. Various pilot projects have been launched in recent years involving several types of nursing profiles, such as APNs or RNs [11–14]. Among these, the MOCCA project (*Nouveau****mo****dèle de****c****oordination en****ca****binet de médecine de famille—*the New Coordination Model for Family Practices) included an RN to perform different primarily fulfil a case management role for patients with chronic illnesses, using individualized care plans [15, 16]. To ensure the development of IP collaboration and case management activities, both impacted by the current reimbursement system [17], the nurses were fully funded by the public health authorities.

In many high-income countries, numerous studies demonstrated that integrating nursing care into PC improves patient satisfaction and care experiences [3, 18–21]. Patient-reported experience measures are commonly used to assess the strengths and weaknesses of healthcare delivery [22]. However, most studies have focussed on APNs, with fewer studies evaluating patients’ experiences of care provided by RNs, though these have shown encouraging results [23, 24]. Additionally, according to the literature, IP collaboration can improve several metrics of the patient experience [25, 26].

The present study assessed patients’ experience of the care provided by IP teams in eight GPs’ practices involved in the MOCCA project across the canton of Vaud, Switzerland. We hypothesized that new nursing activities (especially case management) and the care provided by IP teams would improve patients’ experience of accessibility to care, the comprehensiveness of care, unmet healthcare needs, and care integration.

## Methods

### Study design

Patients’ experience was assessed through a mixed-method study using a convergent design including a quantitative survey using a before-and-after assessment and a qualitative exploratory study [27, 28].

### Setting

The study was conducted between July 2019 and March 2022 in the eight practices participating in the 2-year pilot project MOCCA by integrating an RN. All general practices were located in the canton of Vaud, Switzerland. They were selected on a voluntary basis according to their localization (rural or urban areas) and workforce (number of physicians and medical assistants). Patients’ experience was measured in patients receiving nursing follow-up care with an individualized care plan. As part of this follow-up, nurses provided care to patients with chronic illnesses and carried out case and care management. This included care coordination, therapeutic education, and preventive care.

### Participants in the study

Patients eligible for the quantitative survey were adults over 18 years with a nursing follow-up and individualized care plan and who did not have severe cognitive disorders. The patients’ recruitment was ongoing throughout the pilot project. The project manager monitored the patients’ inclusion and the request for patient’s written informed consents by the GPs.

Eligibility criteria for participating in interviews were the same. Recruitment took place at the end of the first year and at the end of the pilot project. Nurses recruited face-to-face volunteer patients who provided their written informed consent to the GPs. Due to the limited number of patients followed up by nurses and had given their consent, a non-probabilistic, convenience sampling method was used. However, we recruited at least one patient in each general practice.

### Data collection

#### Quantitative data

The overall questionnaire included parts of three validated tools currently used in PC settings. These were the validated French versions of the Population Questionnaire [29, 30], the Patient Experience of Integrated Care Scale [31], and the Person-Centered Primary Care Measure [32]. It also included a section for sociodemographic information, health status, and a satisfaction scale. The full questionnaire is available in Supplementary File 1.

Table 1 summarizes the different dimensions of the three tools used in the questionnaire. We only selected dimensions and items that were applicable in the context of PC in Switzerland and within GPs’ practices.

**Table 1.** Questionnaire composition.

Dimension	Items	Tool
Healthcare use	Section A: questions A1–A3	Population Questionnaire
Unmet healthcare need	Section C: question C1	Population Questionnaire
Accessibility	Section B: questions B1–B9	Population Questionnaire
Care integration	Questions 1–3, 5–7, 9, and 11–17	PEICS (Translation)
Care offered	Section B: questions B28 and B32	Population Questionnaire
Comprehensiveness	All items	PCPCM
Impact of care	Section B: questions B41–B45	Population Questionnaire

The questionnaire was completed before nursing follow-up (T1) and either at the end of the nursing follow-up or the end of the pilot project (T2), but at least 6 months after the beginning of follow-up. The surveys were completed in the general practice using a tablet computer and the REDcap® (Research Electronic Data Capture) tool. To achieve an acceptable response rate, we actively monitored survey responses and sent reminders to nurses to foster participation.

#### Qualitative data

Qualitative data were collected at the end of the project’s first year, varying depending on when practices began the pilot project, between December 2020 and April 2021, and at the end of the 2-year pilot project, between September 2021 and May 2022. A single researcher with a background in both health professions and research conducted face-to-face semi-structured interviews with ten patients. To prove a deeper understanding of the nursing care delivered and its context, these interviews were conducted in the GPs’ consultation rooms immediately following the nursing consultations observed by the researcher. The interviews lasted between 30 to 50 min. To comprehensively explore the dimensions outlined in the questionnaire, the information collected through these interviews included patients’ overall satisfaction with the nursing follow-up care, its impact on their self-empowerment and health, and their experiences regarding care and coordination. The interview guide was developed by the first author and reviewed by the research team. All interviews were audio recorded.

### Data analysis

After verifying the completion of questionnaires both before (T1) and after (T2) the nursing follow-up, the dataset underwent cleaning procedures. Missing data were handled by imputing the mean of the participant’s responses from other questionnaire items. Descriptive statistical analyses were then performed using STATA software (versions 16 and 18) to assess the frequencies, means, and medians of the variables of interest. Additionally, bivariate analyses (*t*-tests) were conducted to compare questionnaire variables between T1 and T2.

Interviews were fully transcribed and identifying data were removed. We used MAXQDA software (V.20 and V.22) to code and analyse the data. For convenience, all qualitative data were coded by a single researcher. Coding data were discussed within the research team and themes were identified. A coding book was established and adapted throughout the analysis process.

### Ethical considerations

This study was conducted in compliance with Swiss law and in accordance with the Declaration of Helsinki. The project was authorized by the Human Research Ethics Committee of the Canton of Vaud (Req-2019-00544). Following their recommendations, written informed consent was obtained by GPs from all the patients included in the study who gave access to health data, questionnaire data, or participated in interviews.

## Results

### Participants and sociodemographic data

Nurses followed up with 438 patients using individualized care plans. Among them, 187 patients gave their written informed consent to participate in the study. A total of 109 patients completed the questionnaires before and after the nursing intervention or at the end of the pilot project. The average participant age was 62 years old, and 62.4% (*n* = 68) were women. 68.8% (*n* = 75) of participants reported one or more diseases, with an average of 2.5 diseases per patient (T1 = 2.48; T2 = 2.49; *P* = .975). 85.3% (*n* = 93) of participants reported taking one or more medications, and the average number of medications taken per participant was 5.3 (T1 = 5.30; T2 = 5.29; *P* = .994) (Table 2).

**Table 2.** Patients’ characteristics.

Characteristics	*N*	*n*	(%)
Patients	109		
Mean age, years, (range)		62 (20–95)	
Sex M F		41 68	37.6 62.4
Patients with diseases Yes Mean number of diseases		75 2.5	68.8
Medication Yes Mean number of medications		93 5.3	85.3

Table 3 presents a comprehensive overview of before-and-after results for each item in the questionnaire, as well as the results of our bivariate analyses. The average interval time between the before (T1) and after (T2) questionnaires was 360 days, ranging from 56 to 788.

**Table 3.** Questionnaire and *t*-test results.

Dimensions	Questionnaire completion time point						Difference *P*^a^
		T1			T2		
	% patients (109)	Mean	95% CI	% patients (109)	Mean	95% CI	
Healthcare use							
Hospitalisation	25.7 (*n* = 109)		17.5–33.9	22.0 (*n* = 109)		14.2–29.8	.525
Use of emergency services	39.5 (*n* = 109)		30.3–48.6	31.2 (*n* = 109)		22.5–39.9	.202
Consultation with a specialist	71.8 (*n* = 103)		63.2–80.5	71.7 (*n* = 106)		63.1–80.3	.981
Consultation with another GP	9.4 (*n* = 106)		3.9–15.0	6.4 (*n* = 109)		1.8–11.0	.413
Average number of visits							
Emergency services		1.8 (*n* = 39)	1.4–2.1		1.7 (*n* = 33)	1.3–2.1	.762
Specialists		4.6 (*n* = 60)	2.3–7.0		4.4 (*n* = 70)	3.1–5.8	.884
Other GPs		2.1 (*n* = 8)	1.2–3.1		2.5 (*n* = 6)	0.9–4.1	.604
Unmet healthcare needs							
Need to see a doctor for a health problem but didn’t see one	17.0 (*n* = 106)		9.8–24.1	3.78 (*n* = 106)		0.2–7.4	.002*
Accessibility							
Appointment							
Same GP (always/often)	95.4 (*n* = 109)		91.5–99.3	95.4 (*n* = 108)		91.4–99.3	.988
Waiting time (< 2 weeks)	81.3 (*n* = 75)		72.5–90.2	91.3 (*n* = 103)		84.7–98.0	.083
Waiting time urgent (< 24 h)	61.9 (*n* = 84)		51.5–72.3	72.0 (*n* = 82)		62.2–81.7	.169
Appointment proposed for < 24 h (always/often)							
Appointment at GP’s practice	63.0 (*n* = 73)		51.9–74.1	71.2 (n = 73)		60.9–81.6	0.291
Return call by GP or nurse	27.9 (*n* = 68)		17.2–38.6	57.5 (*n* = 73)		46.2–68.9	<.001*
See a nurse	24.6 (n = 65)		14.1–35.1	55.6 (*n* = 72)		44.1–67.0	<.001*
See another GP within the practice	23.6 (*n* = 72)		13.8–33.4	26.0 (*n* = 73)		16.0–36.1	.736
See the GP between two other meetings	30.7 (*n* = 62)		19.2–42.1	32.4 (*n* = 71)		21.5–43.3	.829
Go to an emergency department	1.5 (*n* = 65)		0–4.5	8 (*n* = 75)		1.9–14.1	.080
Easy to get an appointment (fully)	86.2 (*n* = 109)		79.8–92.7	84.0 (*n* = 106)		77.0–91.0	.639
Easy to call a GP or nurse (fully)	65.0 (*n* = 97)		55.5–74.4	77.9 (*n* = 104)		69.9–85.9	.04*
Dimensions		Questionnaire completion time point					Difference *P*^a^
		T1			T2		
		Mean	95% CI	% patients	Mean	95% CI	
Integrated care (5-point Likert scale from 0 to 4)							
1. Have all your needs been assessed?		3.74 (*n* = 108)	3.62–3.86		3.81 (*n* = 109)	3.73–3.88	.361
2. Were you as involved as you wanted to be in decisions about your care and support?		3.79 (*n* = 109)	3.68–3.90		3.81 (*n* = 109)	3.70–3.92	.814
3. Was your family or carer as involved in decisions about your care and support as you wanted them to be?		2.70 (*n* = 78)	2.32–3.04		3.00 (*n* = 70)	2.65–3.35	.210
4. Did health and social care staff tell you what will happen next?		3.62 (*n* = 109)	3.46–3.79		3.87 (*n* = 109)	3.77–3.97	.011*
5. When health or social care staff planned care or treatment for you, did it happen?		3.76 (*n* = 109)	3.64–3.88		3.84 (*n* = 109)	3.77–3.92	.249
6. Were your care and support reviewed as they should be?		3.64 (*n* = 107)	3.51–3.77		3.82 (*n* = 108)	3.75–3.90	.014*
7. Did you know who to contact if you needed to ask questions about your condition or treatment?		3.74 (*n* = 108)	3.61–3.87		3.88 (*n* = 108)	3.81–3.95	.056
8. Did health and social care services help you live the life you want?		3.72 (*n* = 109)	3.61–3.85		3.85 (*n* = 107)	3.76–3.94	.104
9. Did health and social care staff give you information about other services that are available to someone in your circumstances, including support organizations?		3.25 (*n* = 106)	3.04–3.46		3.71 (*n* = 107)	3.58–3.84	>.001*
10. Was information provided in a way that you could understand?		3.62 (*n* = 107)	3.47–3.77		3.85 (*n* = 106)	3.77–3.93	.009*
11. Could you meet/phone/email a professional when you needed to ask more questions or discuss the options?		3.06 (*n* = 107)	2.78–3.33		3.71(*n* = 109)	3.58–3.83	>.001*
12. If you still needed contact with previous services/professionals, would it be possible?		2.69 (*n* = 106)	2.40–2.97		2.98 (*n* = 106)	2.70–3.26	.146
13. Did all the different people treating and caring for you work well together to give you the best possible care and support?		3.28 (*n* = 108)	3.09–3.47		3.30(*n* = 108)	3.12–3.48	.888
Dimensions		Questionnaire completion time point					Difference *P*^a^
		T1			T2		
	% patients	Mean	95% CI	% patients	Mean	95% CI	
Care offered							
History	91.7 (*n* = 108)		86.5–96.9	95.4 (*n* = 109)		91.5–99.3	.261
Prevention	81.5 (*n* = 108)		74.2–88.8	90.8 (*n* = 109)		85.4–96.2	.046*
Comprehensiveness (scale from 1 to 4)							
1. My practice makes it easy for me to get care		3.90 (*n* = 109)	3.83–3.96		3.90 (*n* = 109)	3.83–3.96	1.000
2. My practice is able to provide most of my care		3.80 (*n* = 109)	3.71–3.88		3.81 (*n* = 109)	3.74–3.89	.753
3. In caring for me, my doctor considers all of the factors that affect my health		3.83 (*n* = 109)	3.74–3.91		3.89 (*n* = 108)	3.81–3.96	.272
4. My practice coordinates the care I get from multiple places		3.79 (*n* = 108)	3.69–3.88		3.83 (*n* = 109)	3.73–3.92	.563
5. My doctor or practice knows me as a person		3.67 (*n* = 109)	3.52–3.82		3.84 (*n* = 108)	3.76–3.93	.046*
6. My doctor and I have been through a lot together		2.98 (*n* = 108)	2.76–3.20		3.15 (*n* = 108)	2.95–3.34	.266
7. My doctor or practice stands up for me		3.82 (*n* = 108)	3.74–3.91		3.83 (*n* = 109)	3.74–3.91	.979
8. The care I get takes into account knowledge of my family		3.62 (*n* = 108)	3.48–3.76		3.69 (*n* = 106)	3.56–3.83	.470
9. The care I get in this practice is informed by knowledge of my community		3.44 (*n* = 105)	3.24–3.63		3.62 (*n* = 106)	3.45–3.79	.156
10. Over time, this practice helps me to meet my goals		3.62 (*n* = 107)	3.48–3.76		3.72 (*n* = 109)	3.62–3.83	.215
11. Over time, my practice helps me stay healthy		3.74 (*n* = 106)	3.63–3.84		3.80 (*n* = 109)	3.70–3.89	.391
Impact of care (scale from 1 to 4)							
1. Better understanding of health problem		3.88 (*n* = 107)	3.81–3.95		3.91 (*n* = 108)	3.85–3.97	.532
2. Preventing health problems		3.68 (*n* = 103)	3.55–3.81		3.83 (*n* = 108)	3.74–3.92	.047*
3. Better control of health problems		3.76 (n = 106)	3.67–3.86		3.84 (*n* = 109)	3.76–3.93	.216
4. Support in taking medication		3.93 (*n* = 107)	3.86–3.99		3.96 (*n* = 108)	3.92–4.00	.332
5. Support in adopting healthy lifestyle habits		3.72 (*n* = 105)	3.61–3.84		3.88 (*n* = 108)	3.80–3.96	.024*
Satisfaction (scale from 0 to 10)		9.08 (*n* = 109)	8.83–9.33		9.17 (*n* = 109)	8.89–9.46	.631
Self-perceived health (scale from 0 to 10)		6.53 (*n* = 108)	6.22–6.83		6.92 (*n* = 109)	6.57–7.27	.097

^*^significant p-value

Analyses showed that integrating nurses into GPs’ practices significantly impacted patients’ experience of unmet healthcare needs. Specifically, the percentage of patients reporting unmet needs (defined as needing to see a GP without having seen one) decreased significantly from 17.0% to 3.8% (*P* = .002) with nursing follow-up. The results also showed that nurses’ involvement in patient follow-up enhanced several aspects of accessibility. The ability to meet urgent appointment needs improved, evidenced by a significant increase in return telephone calls from a GP or nurse (T1 = 27.94%; T2 = 57.53%; *P* < .001) and the availability of nurse consultations (T1 = 26.62%; T2 = 55.56%; *P* < .001). Patients also found it easier to access their GP or a nurse by telephone (T1 = 65.0%; T2 = 77.9%; *P* = .04). However, there were no significant differences regarding other aspects of accessibility.

Regarding integrated care, all items measured on a 5-point Likert scale (from 0 to 4) showed improvements in nurse involvement. Significant differences were found in five items: information on the next steps in care (T1 = 3.62; T2 = 3.87; *P* = .011), review of care and support (T1 = 3.64; T2 = 3.82; *P* = .014), information from professionals about other available services, including support organizations (T1 = 3.25; T2 = 3.71; *P* > .001), understandable information (T1 = 3.62; T2 = 3.85; *P* = .009), and the ability to contact a health professional if needed (T1 = 3.06; T2 = 3.71; *P* > .001). Care’s comprehensiveness also increased with the presence of nurses and significantly so for the items regarding the patient’s overall knowledge (T1 = 3.67; T2 = 3.84; *P* = .046).

Furthermore, nurses significantly enhanced the quality of preventive care. The percentage of patients who felt that healthcare professionals took the time to talk to them about prevention increased significantly, from 81.48% to 90.83% (*P* = .046). This positive trend was also observed in the prevention of health problems (T1 = 3.68; T2 = 3.83; *P* = .047) and support for adopting healthy lifestyle habits (T1 = 3.72; T2 = 3.88; *P* = .024).

However, despite improvements in these different dimensions of the patients’ experience, nursing care did not significantly affect overall healthcare use. There were no significant changes in the percentage of patients hospitalized (T1 = 25.7%; T2 = 22.0%; *P* = .525), those who used emergency services (T1 = 39.5%; T2 = 31.2%; *P* = .202) or those who visited a specialist (T1 = 71.8%; T2 = 71.7%; *P* = .981) or other GP (T1 = 9.4%; T2 = 6.4%; *P* = .413). Lastly, nurses’ presence did not significantly improve patients’ self-perceived health based (T1 = 6.53; T2 = 6.92; *P* = .097) or their overall satisfaction (T1 = 9.08; T2 = 9.17; *P* = .631).

### Qualitative results from interviews

The following qualitative results came from interviews with seven women and three men. The average age of patients interviewed was 61 years old. Several major themes emerged from these interviews, including nurses substituting for GPs, nursing accessibility and availability, and the impact of nursing care and care coordination.

#### Nurses substituting for GPs

Patients perceived an appointment with a nurse to be a good alternative to a GP, especially in less critical situations or for routine monitoring. As one patient said, (P1) *‘It makes me feel safer. Because sometimes, […], I hesitate, because I tell myself that it [my health status] has to be worse to go and see the GP. I feel like I have an intermediary’.* Having nurses substituting for GPs was well perceived, with patients estimating that nursing care was of as good quality as medical care: (P2) ‘*For me, she does it as well as the GP’.*

#### Nursing accessibility and availability

Nurses seemed more accessible and available to patients than did GPs, with whom they did not have the time to discuss all their health problems, as one patient remarked: (P9) *‘I go through the nurse because I know that with her, I’ll have an appointment quickly [...] Then, with her, the nurse, I have more time to make a strategic plan. I couldn’t spend 1 hour at a time meeting with the doctor’*. Patients also mentioned that it was easier for them to ask nurses questions: (P8) ‘*I had more information from the nurse, but I didn’t dare question the doctor’.*

#### Impact of nursing care and care coordination

Nurses were also very good at re-explaining things or supplementing information previously given by the GP, as one patient explained: (P7) *‘She also helps me because sometimes, with doctors, I don’t understand them. But she also explains things better, in her own way’.*

Patients also felt well listened to and included in decisions concerning their health: (P2) *‘They don’t make decisions without asking me..., without getting my opinion, or they always ask me what it is I want’.* Patients found relationships and consultations with nurses to be agreeable: (P8) ‘*The GP is really the specialist, finding out about your illness and advising you to take medication, but with the nurse, it’s more like having a chat. You feel like a member of the family. It’s heartwarming’.* These findings highlighted that nursing skills were very important to patients.

#### Nursing support and follow-up

Nursing support and follow-up also had an impact on patients’ lifestyle habits: (P7) *‘When I came back [to see the nurse], I had a completely different attitude. Things were better; I was more careful about what I ate’.* For some patients, nursing follow-up also affected their state of health, as one patient stated: (P9) *‘But, it’s true that without the nurse, I wouldn’t be where I am today, with clean coronary arteries and weight loss, too’.* Findings also confirmed that nurses had increased the opportunities for preventive care. Interviews highlighted how nurses managed mental health problems, too: (P4) *‘I think that if I hadn’t had the nurse, for a session that helped me, then I think I would have made another appointment with the psychiatrist, which I didn’t do, and I got out of the whole thing [with the only support of the nurse]’.* Nurses also addressed patients’ social concerns, as this patient explained: (P3) *‘She intervened with the community healthcare centre, explaining that I really needed transport for an operation and everything’.*

#### Information exchange among healthcare professionals

Lastly, patients expressed their satisfaction with how healthcare professionals had exchanged information regarding their health. The fact that they worked in the same practice and could exchange information face-to-face was also perceived as reassuring by patients: (P8) *‘He has direct communication with this nurse, because they are in the same practice […]. They have more information, and the nurse can look at my file right away. That’s reassuring’.* Overall, patients were fully satisfied with in-practice nursing follow-up. One patient mentioned that thanks to nursing follow-up, it was (P1) *‘a great bonus to feel better when I leave’.*

## Discussion

Findings indicated that involving nurses in case management activities only improved a few items of the patient’s experience. Bivariate analyses revealed significant improvements in specific dimensions related to unmet care needs, access to care facilitated by nurses, preventive healthcare services, and the quality of information provided by healthcare professionals in GP practices. Qualitative data supported these findings, emphasizing nurses’ availability and ability, comprehensive care, as well as complementary, and easier-to-understand information.

In agreement with the existing literature, integrating nurses into general practices and the development of IP collaboration contributed to enhancing preventive care and patient education by complementing and enriching their understanding of health-related information [9, 33–35] and by addressing some patients’ previously unmet needs [36, 37]. Therefore, by providing more comprehensive care, nurses strengthened the overall impact of care, particularly in terms of initiating changes in lifestyle habits, and preventing health problems. These various dimensions contributed to enhancing patients’ health and well-being, as observed during interviews with patients and supported by the existing literature [34].

Furthermore, the holistic care provided by nurses in collaboration with GPs, which was able to comprise psychosocial elements of care, also played a role in improving well-being. Although not intended to replace social workers or psychologists, patient-centred holistic nursing care is suitable for patients with chronic conditions and at a high risk of psychosocial issues resulting from chronic illnesses [38]. The literature review conducted by Halcomb et all also shows that nurses can play a role in the management of mental health problems, improving outcomes [39].

Additionally, our findings revealed that integrating case management activities into general practices reduced unmet healthcare needs. Indeed, the accessibility and availability of nursing services appeared to successfully substitute for the need to see a GP in some situations, as well as reducing unmet needs. More comprehensive care, encompassing prevention and care coordination, could indeed reduce unmet healthcare needs and subsequently reduce healthcare use among frequent users [40, 41]. In this way, nursing follow-up, with a specific range of activities, could represent a valuable addition to overburdened GPs’ practices, potentially easing some of the demand for consultations with GPs, particularly among frequent users, which is a recurring problem in Switzerland [42]. However, the development of nursing activities in the MOCCA pilot projects and the accessibility and availability of nurses were only made possible because the nurses were fully financed by the canton of Vaud’s public health authorities. Indeed, Switzerland’s healthcare provision reimbursement system limits payments for prevention and coordination activities, especially when performed by nurses.

As our quantitative and qualitative findings highlighted, nurses provide new opportunities for improving the range of and access to PC services in GPs’ practices; they also shorten the time between a healthcare need and access to a healthcare professional. Integrating nursing care into the follow-up for PC patients with chronic illness reinforces access to care [20, 23]. Improving care accessibility depends principally on nurses’ roles and activities. An extended range of roles seems to improve care accessibility more [23].

Switzerland often ranks well in international comparisons of healthcare accessibility [43]. However, more recently, there has been a decline in accessibility due to an ongoing shortage of GPs and increasing numbers of chronically ill patients [10, 44]. Finding a GP, especially in suburban areas, is becoming challenging in Switzerland. In this context, nurses can reduce GPs’ workloads, thereby freeing up some time to maintain their accessibility [20, 23] for existing and new patients. Additionally, substituting GPs with nurses is generally well-received by patients [18, 45], as our results also indicated. Nursing care could, therefore, also support healthcare systems by optimizing medical care use.

### Limitations

We faced statistical challenges due to a restricted sample size of patients who completed the before-and-after questionnaire. This low response rate was primarily because nurses proposed the questionnaire to patients and were reluctant to do it. Consequently, we were unable to conduct multivariate analyses that could have included general practices as variables, hindering our ability to identify the impacts of care organization and resources on nurses’ ranges of activities and their consequences on patients’ experience [23, 46]. Moreover, our evaluation was restricted to a before-and-after comparison without a control group. The absence of a control group makes it difficult to distinguish intervention effects from a secular trend or variations linked to causes other than the intervention itself. Additionally, lacking information on non-respondents to the questionnaire could also introduce bias. In terms of qualitative data, potential selection bias cannot be discounted, as all participants had agreed to receive follow-up care from a nurse. Lastly, there is a possibility of desirability bias in both qualitative and quantitative data, where respondents may provide socially desirable responses rather than their true experiences or opinions.

## Conclusion

Integrating RNs and new case management activities as part of interprofessional teams in GPs’ practices could be appropriated for addressing patients’ unmet care needs, promoting preventive care, improving patients’ understanding of health issues, and supporting patients in changing their lifestyle habits.

## Supplementary Material

cmaf011_suppl_Supplementary_Files_1

## Data storage and availability

The data are stored at the University of Lausanne’s Center for Primary Care and Public Health. Datasets generated and analysed during the current study are not publicly available. At this point, researchers are still working on the material; however, it is available from the corresponding author upon reasonable request.
