# Supplementary material for: Patients’ experience of integrated nursing care in general practices in Switzerland: a mixed-methods study
Source: Fam Pract. 2025 Mar 19;42(2):cmaf011. doi: 10.1093/fampra/cmaf011 (PMC11920863; doi:10.1093/fampra/cmaf011)
Supplement: cmaf011_suppl_Supplementary_Files_1 [file cmaf011_suppl_supplementary_files_1.pdf]

Supplementary material 1: Questionnaire

ID ---------------------------
Date: ……/……/……

DONNÉES DÉMOGRAPHIQUES :

1-Êtes-vous un.e ? □ Homme □ Femme

2-Quelle est votre année de naissance ? ……./……/……..

3-Quel est votre lieu de naissance ? : ………….

□ Suisse

□ Dans un autre pays de l'Union Européenne

□ Dans un pays européen en dehors de l'union Européenne

□ Dans un autre pays

4-Vivez-vous ?

□ Seul(e) □ Seul(e) avec enfant (s) □ En couple avec enfant (s)

□ En couple sans enfant □ Avec vos parents □ Je ne souhaite pas répondre

RECOURS AUX SOINS :

5-Au cours de la dernière année, combien de fois avez-vous été hospitalisé (e) c'est-à-dire avoir passé au moins une nuit à l'hôpital ?

□ 0 0 fois

□ 1 1 fois

□ 2 2 fois

□ 3 3 fois ou plus

□ 8 Je ne sais pas / je ne me souviens pas

6-Au cours de la dernière année, combien de fois vous êtes-vous présenté aux urgences d'un hôpital pour recevoir des soins médicaux?

□ 0 0 fois

□ 1 1 fois

□ 2 2 fois

□ 3 3 fois

□ 4 4 fois

□ 5 5 fois ou plus

□ 6 Je ne sais pas / je ne me souviens pas

7-Avez-vous consulté un médecin spécialiste ?

□ 1 Oui

□ 2 Non

□ 3 Je ne sais pas / je ne me souviens pas

8-Si oui, combien de fois avez-vous consulté? □ ….. □ 2 Je ne sais pas / je ne me souviens pas

9-Avez-vous consulté d'autres médecins généralistes que le vôtre?

□ 1 Oui

□ 2 Non

□ 3 Je ne sais pas / je ne me souviens pas

10-Si oui, combien de fois avez-vous consulté? □ …… □ 2 Je ne sais pas / je ne me souviens pas

LES BESOINS NON COMBLES

11-Au cours de la dernière année, avez-vous ressenti le besoin de consulter un médecin sans en avoir vu un ?

□ 1 oui

□ 2 non

□ 8 je ne sais pas/ je ne me souviens pas

L'ACCESSIBILITÉ

12-Quand vous venez consulter dans ce cabinet, voyez-vous le même médecin ?

□ 1 Toujours

□ 2 Souvent

□ 3 Parfois

□ 4 Jamais

□ 5 Je ne sais pas / je ne me souviens pas

□ 6 C'est la première fois que je viens

13-Lorsque vous avez besoin de voir votre médecin dans ce cabinet en général combien de temps cela prend-il pour le rencontrer?

□ 1 moins de 2 semaines

□ 2 de 2 à 4 semaines

□ 3 de 1 à 3 mois

□ 4 4 mois et plus

□ 5 rendez-vous toujours préfixés

□ 6 ne prend jamais de rendez-vous *passez à la question 15*

□ 8 Je ne sais pas / je ne me souviens pas

14-Diriez-vous que ce délai pour voir votre médecin sur rendez-vous est….

□ 1 très long

□ 2 long

□ 3 court

□ 4 très court

□ 8 Je ne sais pas / je ne me souviens pas

15-Lorsque vous avez besoin de soins immédiats ou urgents, combien de temps cela prend-il pour voir un médecin à ce cabinet ?

□ 1 moins de 24 heures

□ 2 de 1 à 2 jours

□ 3 de 3 à 4 jours

□ 4 5 jours ou plus

□ 8 Je ne sais pas / je ne me souviens pas

16-Au cours de cette dernière année, avez-vous contacté le cabinet pour obtenir des soins le jour même ou le lendemain alors que vous n'aviez pas rendez-vous?

□ 1 oui

□ 2 non

□ 3 Je ne sais pas / je ne me souviens pas

17-Est-ce que dans ces situations, on vous propose ….

		Toujours	Souvent	Parfois	Jamais	Je ne sais pas /je ne me souviens pas
A	de vous présenter directement au cabinet					
B	Qu'un médecin ou un.e infirmier-ère vous rappelle					
C	De venir voir l'infirmier-ère du cabinet					
D	De venir voir un autre médecin					
E	De venir voir ?votre médecin entre deux rendez-vous					
F	De vous présenter à un autre cabinet sans rendez-vous ou aux urgences					

18-Nous aimerions maintenant connaître votre opinion sur l'accessibilité du cabinet. Pour chacune des affirmations suivantes, dites-nous si vous êtes beaucoup, moyennement, un peu ou pas du tout en accord.

		Beaucoup en accord	Moyennement	Un peu	Pas du tout en accord	Je ne sais pas /je ne me souviens pas
A	Il est facile d'obtenir un rendez-vous à ce cabinet					
B	Il est facile de parler à un médecin ou à un.e infirmie-èr-e par téléphone à cet endroit lorsque c'est ouvert					

INTEGRATION DES SOINS

En pensant aux soins que vous avez reçus au cours des 12 derniers mois…

19-Vos besoins ont-ils tous été évalués par les professionnels de la santé?

☐ Tous mes besoins ont été évalués

☐ La plupart de mes besoins ont été évalués

☐ Certains de mes besoins ont été évalués

☐ Peu de mes besoins ont été évalués

☐ Aucun de mes besoins n'a été évalué

☐ Je ne sais pas / ne me rappelle pas

20-Avez-vous été impliqué autant que vous le souhaitiez dans les décisions au sujet de vos soins de santé?

☐ Toujours

☐ Souvent

☐ Parfois

☐ Rarement

☐ Jamais

21-Votre famille ou proche a-t-il été impliqué dans les décisions de votre santé autant que vous le souhaitiez?

☐ Toujours

☐ Souvent

 Parfois

 Rarement

 Jamais

 Je n’ai pas de famille ou proche aidant disponibles pour s’impliquer
 Je ne veux pas ou je n’ai pas besoin que ma famille ou mon proche aidant soit impliqué dans les décisions au sujet de mes soins de santé

22-Les professionnels de la santé vous ont-ils informé des prochaines étapes de vos soins?

 Toujours

 Souvent

 Parfois

 Rarement

 Jamais

23-Avez-vous reçu les soins ou les traitements que des professionnels de la santé avaient planifié pour vous?

 Toujours

 Souvent

 Parfois

 Rarement

 Jamais

En pensant aux soins et que vous avez reçus au cours des 12 derniers mois…

24-Vos soins de santé ont-ils été réévalués comme vous croyez qu’ils devaient l’être?

 Toujours

 Souvent

 Parfois

 Rarement

 Jamais

25-Saviez-vous qui contacter si vous aviez besoin de poser des questions sur votre condition ou votre traitement?

 Toujours

 Souvent

 Parfois

 Rarement

 Jamais

26-Les différentes personnes qui vous ont traité et ont pris soin de vous ont-elles bien travaillé ensemble afin de vous donner les meilleurs soins et soutien possibles?

 Toujours

 Souvent

 Parfois

 Rarement

 Jamais

27-Les professionnels de la santé vous ont-ils aidé à vivre la vie que vous vouliez?

 Énormément

 Beaucoup

 Moyennement

 Un peu

 À peu près pas

28-Les professionnels de la santé vous ont-ils donné de l'information sur d'autres services disponibles, incluant des organismes de soutien?

 Toujours

 Souvent

 Parfois

 Rarement

 Jamais

En pensant aux soins que vous avez reçus au cours des 12 derniers mois…

29-L'information vous a-t-elle été fournie de manière compréhensible?

 Toujours

 Souvent

 Parfois

 Rarement

 Jamais

30-Avez-vous pu contacter un professionnel de la santé lorsque vous aviez besoin de poser plus de questions ou de discuter des options de soin?

- ☐ Toujours
- ☐ Souvent
- ☐ Parfois
- ☐ Rarement
- ☐ Jamais

31-A-t-il été possible pour vous de prendre contact avec des professionnels de la santé vus dans le passé si vous en aviez besoin?

- ☐ Toujours
- ☐ Souvent
- ☐ Parfois
- ☐ Rarement
- ☐ Jamais

32-Les professionnel-le-s de santé vous ont-ils aidé à vivre la vie que vous vouliez ?

- ☐ Enormément
- ☐ Beaucoup
- ☐ Moyennement
- ☐ Un peu
- ☐ A peu près pas

L'OFFRE DES SOINS

33-Toujours en considérant votre expérience de soins lors de votre dernière année, dites-nous si vous êtes beaucoup, moyennement, un peu ou pas du tout en accord avec les énoncés suivants :

Dans ce cabinet…..		Beaucoup en accord	Moyennement	Un peu	Pas du tout en accord	Ne sait pas / ne se souvient pas
	Votre histoire médicale est connue (vos maladies antérieures)					
	Lors de vos visite, l'équipe de soin prend-elle le temps de vous parler de prévention et vous interroge sur vos habitudes de vie					

LA PRISE EN CHARGE :

34-En considérant la prise en charge dont vous avez bénéficié dans ce cabinet lors de l'année écoulée, dites-nous dans quelle mesure vous êtes d'accord avec les énoncés suivants

	Tout à fait	Le plus souvent	Parfois	Pas du tout
Ce cabinet fait en sorte que je puisse obtenir des soins facilement				
Ce cabinet assure la plupart des soins dont j'ai besoin				
Pour ma prise en charge, mon médecin considère l'ensemble des éléments qui influencent ma santé				
Ce cabinet coordonne les soins dont j'ai besoin avec les différents partenaires				
Mon médecin ou mon cabinet me connait en tant que personne				
Mon médecin et moi avons traversé beaucoup de choses ensemble				
Mon médecin ou mon cabinet me soutient				
Les soins que je reçois tiennent compte de mon environnement familial				
Les soins que je reçois dans ce cabinet prennent en compte la communauté à laquelle j'appartiens				
Ce cabinet m'aide à atteindre mes objectifs au fil du temps				
Ce cabinet m'aide à rester en bonne santé au fil du temps.				

IMPACT DES SOINS :

35-En considérant les soins que vous avez reçus au cabinet, dites-nous si vous êtes beaucoup, moyennement, un peu ou pas du tout en accord avec les énoncés suivants….

		Beaucoup en accord	Moyennement	Un peu	Pas du tout en accord	Je ne sais pas / ne me se souviens pas
La prise en charge que vous recevez à cet endroit vous permet	de mieux comprendre vos problèmes de santé					
	de prévenir certains problèmes de santé avant qu'ils ne surviennent					
	de bien contrôler vos problèmes de santé					

	Les professionnel-le-s que vous voyez à cet endroit vous encouragent à suivre les traitements prescrits					
	Les professionnel.le.s que vous voyez à cet endroit vous aident à vous motiver à adopter de bonnes habitudes de vie comme cesser de fumer, mieux manger					

Données personnelles

36-Comment décrirez-vous votre occupation actuelle ou votre statut professionnel ?

□ En emploi

□ Indépendant (e)

□ Etudiant (e)

□ En recherche d’emploi

□ Dans l’incapacité de travailler du fait d’une maladie ou d’un handicap

□ Retraité (e)

37-Quel est votre plus haut niveau de scolarité atteint ?

□ Scolarité obligatoire

□ Enseignement secondaire (Baccalauréat, CFC)

□ Enseignement supérieur (Université, Haute Ecole)

État de santé

38-En général, diriez-vous que votre santé est...

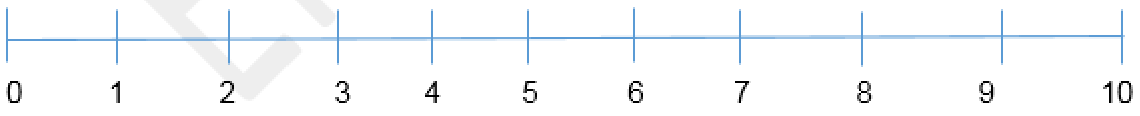

Indiquez avec une croix sur cette échelle de 0 à 10 où vous situez votre état de santé global, 10 étant le meilleur état de santé possible et 0 le moins bon

39-Avez- vous une ou plusieurs maladies chroniques (maladie de longue durée comme l’hypertension artérielle, le diabète, la dépression, l’asthme…)

□ 1 oui

□ 2 non

Si oui combien de différentes maladies de ce type avez-vous ? …………………………

40-Prenez-vous régulièrement un ou plusieurs médicaments sur ordonnance ?

□ 1 oui

□ 2 non

Si oui, combien de différents médicaments sur ordonnance prenez-vous régulièrement et de façon continue? ……………………………………………

41-Globalement, comment êtes-vous satisfait de votre prise en charge dans le cabinet médical ?

0 1 2 3 4 5 6 7 8 9 10

Indiquez avec une croix sur cette échelle de 0 à 10 où vous situez votre satisfaction, 10 étant la meilleure et 0 le moins bon

MERCI DE VOTRE PARTICIPATION
